# Vaniprevir plus peginterferon alfa-2b and ribavirin in treatment-naive Japanese patients with hepatitis C virus genotype 1 infection: a randomized phase III study

**DOI:** 10.1007/s00535-015-1120-x

**Published:** 2015-09-25

**Authors:** Norio Hayashi, Makoto Nakamuta, Tetsuo Takehara, Hiromitsu Kumada, Akiko Takase, Anita Yee Mei Howe, Steven W. Ludmerer, Niloufar Mobashery

**Affiliations:** Kansai Rosai Hospital, 1-69 Inabasou 3-chome, Amagasaki, Hyogo 660-8511 Japan; Department of Gastroenterology, National Hospital Organization Kyushu Medical Center, 1-8-1 Jigyohama, Chuo-Ku, Fukuoka, 810-8563 Japan; Department of Gastroenterology and Hepatology, Osaka University Graduate School of Medicine, 2-2 Yamadaoka, Suita, Osaka 565-0871 Japan; Department of Hepatology, Toranomon Hospital, 1-3-1 Kajigaya, Takatsu-ku, Kawasaki, 213-8587 Japan; MSD K.K., 1-13-12 Kudan-kita, Chiyoda-Ku, Tokyo, 102-8667 Japan; Merck & Co., Inc., 2000 Galloping Hill Road, Kenilworth, NJ 07033 USA

**Keywords:** Vaniprevir, Hepatitis C virus, Peginterferon, Ribavirin, Japan

## Abstract

**Background:**

Vaniprevir is a potent macrocyclic hepatitis C virus (HCV) nonstructural protein 3/4A protease inhibitor. This phase III study evaluated the safety and efficacy of vaniprevir in combination with peginterferon alfa-2b and ribavirin (PR) for 24 weeks compared with PR alone for 48 weeks in treatment-naive Japanese patients with HCV genotype 1 infection.

**Methods:**

Treatment-naive Japanese patients with HCV genotype 1 infection were randomly assigned to receive vaniprevir (300 mg twice daily) plus PR for 12 weeks then PR alone for 12 weeks, vaniprevir (300 mg twice daily) plus PR for 24 weeks, or PR alone for 48 weeks. The primary end point was sustained virologic response 24 weeks after completion of treatment (SVR_24_).

**Results:**

In total, 294 patients were randomly assigned to receive treatment. Most patients had HCV genotype 1b infection (98 %, 288 of 294 patients). SVR_24_ was achieved in 83.7, 84.5, and 55.1 % of the patients in the vaniprevir 12-week, vaniprevir 24-week, and control arms, respectively. The difference in SVR_24_ rates between each vaniprevir arm and the control arm was statistically significant (*p* < 0.001 for both). Relapse was commoner in the control arm (29.5 %) than in the vaniprevir arms (8.6 % and 10.5 % for the 12-week and 24-week arms, respectively). Commonly reported adverse events were generally similar across treatment arms, with the exception of an increase in the incidence of gastrointestinal adverse events such as nausea, diarrhea, and vomiting in patients receiving vaniprevir. These events were considered manageable.

**Conclusion:**

Vaniprevir is a valuable addition to the therapeutic options available to Japanese patients with HCV genotype 1 infection who are eligible for interferon-based treatment.

**ClinicalTrials.gov identifier:**

NCT01370642.

**Electronic supplementary material:**

The online version of this article (doi:10.1007/s00535-015-1120-x) contains supplementary material, which is available to authorized users.

## Introduction

There are approximately two million patients with hepatitis C virus (HCV) infection in Japan [1]. HCV infection is the leading cause of hepatocellular carcinoma in Japan, leading to more than 30,000 deaths each year. Peginterferon and ribavirin dual therapy has improved sustained virologic response (SVR) rates for patients with HCV infection; however, patients with HCV genotype 1 infection still experience virologic failure with this treatment.

Direct-acting antiviral agents (DAAs) have revolutionized the treatment of chronic HCV infection. Compared with peginterferon and ribavirin dual therapy, regimens including a DAA offer a greater opportunity for viral eradication, often achieving substantially higher efficacy with shorter treatment durations. Indeed, interferon-free regimens are now becoming available in certain geographic regions [2, 3]. However, from public health and health equity perspectives, there is an urgent need to overcome the numerous barriers to care and treatment for HCV infection in resource-constrained areas [4].

In Japan, the DAAs telaprevir and simeprevir were approved in 2011 and 2013, respectively, as components of triple therapy regimens in patients who have HCV genotype 1 infection with high viral load [5–9]. Both agents, in combination with peginterferon and ribavirin, yield improved SVR rates compared with peginterferon and ribavirin alone; however, telaprevir is limited by an increased incidence of anemia and serious skin rashes [8]. In addition, dual oral DAA therapy with daclatasvir and asunaprevir [10] was approved in Japan in July 2014.

Vaniprevir is a potent macrocyclic HCV nonstructural protein (NS) 3/4A protease inhibitor [11] that exhibits pronounced antiviral activity in vitro and in vivo [12, 13] and has demonstrated antiviral efficacy in combination with peginterferon and ribavirin in several phase II clinical trials in treatment-naive and treatment-experienced patients with HCV genotype 1 infection [14–17]. On the basis of the cumulative efficacy, safety, and pharmacokinetic data, vaniprevir at a dosage of 300 mg twice daily was selected for further evaluation [17]. Phase III studies of vaniprevir have been conducted in treatment-naive patients with HCV genotype 1 infection and in patients with HCV genotype 1 infection who relapsed or were nonresponders following prior treatment with interferon-based therapy. Herein we present the results of a phase III study in treatment-naive patients that evaluated the safety and efficacy of vaniprevir (300 mg twice daily) plus peginterferon alfa-2b and ribavirin (PR) for 24 weeks compared with PR alone for 48 weeks in treatment-naive Japanese patients with HCV genotype 1 infection. Vaniprevir plus PR received marketing approval in Japan in September 2014. For patients with HCV genotype 1 infection and high viral load, the Japan Society of Hepatology “Guidelines for the management of hepatitis C virus infection (ver 3.4)” recommend that simeprevir or vaniprevir plus PR is considered as first-line therapy for treatment-naive patients who are eligible for interferon-based therapy, and that dual oral therapy with daclatasvir and asunaprevir be considered in treatment-naive patients who are not eligible for interferon-based therapy.

## Methods

The study was conducted in accordance with principles of good clinical practice, and was approved by the appropriate institutional review boards and regulatory agencies, and is registered with ClinicalTrials.gov (identifier NCT01370642, protocol 043).

### Patients

Japanese patients aged 20–70 years with chronic, compensated HCV genotype 1 infection were enrolled in the study. Other key inclusion and exclusion criteria included no history of interferon-based antiviral therapy, no evidence of cirrhosis, HCV RNA levels of 5.0 log IU/mL or greater, and other protocol-defined laboratory values at screening. Patients with HIV or hepatitis B virus infection or evidence of chronic hepatitis because of a non-HCV-related cause were excluded.

### Study design

This was a phase III, multicenter, randomized, placebo-controlled study. The study was double-blinded up to the week 24 visit; after this visit, patients, investigators, and personnel employed by the study sponsor were inevitably unblinded because of the difference in the durations of the treatment period among the study arms (24 weeks for vaniprevir plus PR; 48 weeks for PR alone). The official unblinding was performed after the data has been declared complete and protocol violations had been identified for all visits in all patients for analysis of the primary end point.

Patients were randomly assigned 1:1:1 to one of three treatment arms. In arm 1 (hereinafter referred to as the 12-week arm), patients received vaniprevir (300 mg twice daily) plus PR [peginterferon alfa-2b (1.5 µg/kg/week) and ribavirin (600–1000 mg/day)] for 12 weeks followed by placebo plus PR for 12 weeks (total treatment duration 24 weeks); in arm 2 (hereinafter referred to as the 24-week arm), patients received vaniprevir (300 mg twice daily) plus PR for 24 weeks (total treatment duration 24 weeks); and in arm 3 (hereinafter referred to as the control arm), patients received placebo plus PR for 24 weeks, then PR alone for 24 weeks (total treatment duration 48 weeks). To maintain blinding, vaniprevir and identically appearing placebo capsules were prepared centrally and supplied to the investigators. Randomization was stratified according to age (younger than 65 years/65 years or older), site, and *IL28B* (rs12979860) genotype (major CC/minor CT and TT). Randomization was performed by a computer-generated randomized allocation schedule prepared by the study sponsor, independently of the study team, and implemented by a third-party vendor. Dose reduction and interruption of PR treatment were permitted as defined in the protocol. Vaniprevir dose adjustment was not permitted. Adherence was calculated as [total administered dose for each medication/(dose defined by protocol × defined treatment days)] × 100 (%).

Virologic failure was defined as detectable HCV RNA at treatment week 36 (applicable for the control arm only), virologic breakthrough (undetectable HCV RNA followed by an HCV RNA level greater than 1000 IU/mL while the patient was receiving therapy), incomplete virologic response/rebound (≥1 log increase in HCV RNA level from the nadir followed by an HCV RNA level greater than 1000 IU/mL), or relapse (detectable HCV RNA at two consecutive visits following the end of all study treatment after the patients has had undetectable HCV RNA while receiving treatment). For patients in the control arm with virologic failure, treatment was discontinued, and after reconfirmation of eligibility and their reconsent, they were offered further open-label treatment with vaniprevir plus PR for 24 weeks (rollover arm).

### End points

The primary efficacy end point was SVR_24_, defined as undetectable HCV RNA 24 weeks after completion of treatment. Secondary virologic end points included the proportion of patients with rapid virologic response (undetectable HCV RNA at treatment week 4), complete early virologic response (undetectable HCV RNA at treatment week 12), end-of-treatment response, and SVR_12_ (undetectable HCV RNA 12 weeks after completion of treatment). Safety evaluations included adverse event (AE) reporting, laboratory test values, physical examinations, 12-lead electrocardiography (ECG), and vital sign assessments. Safety events prespecified as events of interest in the protocol were rash categorized as a serious AE (SAE), anemia (anemia and hemoglobin decreased), neutropenia (neutropenia and neutrophil count decreased), blood bilirubin increased, and gastrointestinal AEs (vomiting, nausea, and diarrhea). Resistance-associated variants (RAVs) in the HCV NS3 region were evaluated as one of the exploratory end points defined in the protocol. Baseline samples were tested for RAVs in all patients. Additional samples from patients who met the criteria for virologic failure were tested for RAVs at the time of failure (or with the first sample collected following failure) and for an additional follow-up period as defined in the protocol. Additional testing for variants in the HCV NS5A region was retrospectively conducted as part of exploratory research, with use of plasma samples collected at the baseline and at the time of virologic failure from patients with HCV genotype 1b infection who consented to optional specimen collection for future biomedical research.

### Assays

#### Serum HCV RNA concentrations

Serum HCV RNA levels were measured with the Roche COBAS^®^ TaqMan^®^ HCV auto assay. The limit of quantification was 1.2 log IU/mL (15 IU/mL) and the limit of detection was less than 1.2 log IU/mL, but with no specific value.

#### Resistance testing

RAVs in the NS3/4A gene and the NS5A gene were assessed by the direct sequencing method (Sanger method/population sequencing method). Assessment of RAVs in the NS3/4A and NS5A regions was performed only in samples with a viral titer greater than 1000 IU/mL because of the sensitivity of the assay. The HCV NS3/4A gene was amplified, population sequenced, and compared with the respective reference sequence, GT1a_H77 (GenBank AF009606) for genotype 1a, or GT1b_Con1 (GenBank AJ238799) for genotype 1b to identify polymorphisms at each amino acid position. Resistance analysis focused on amino acid polymorphisms that have previously been detected in patients in whom treatment with HCV protease inhibitors, including vaniprevir, failed. These variants encompass amino acid residues V36, Q41, F43, T54, V55, Y56, Q80, R155, A156, D168, I170 (genotype 1a), and V170 (genotype 1b) within the NS3 protease domain. In addition, amino acid residues L23, Q24, L28, R30, L31, P32, F37, Q54, P58, Q62, A92, and Y93 were included in the analysis of variants in the NS5A gene. The NS3/4A and NS5A evaluations were performed independently, and the sensitivities of the assays are such that a given polymorphism must be present in at least 25 % (NS3/4A) or 10 % (NS5A) of the total viral population to be detected. In vitro potency measurements were made with the replicon system as previously described [12]; vaniprevir potency was measured in triplicate in a 20-point twofold dilution series over a concentration range of 0.019 nM to 10 μM. Mutants within the NS3 gene or NS5A gene were engineered into the GT1a_H77 or GT1b_Con1 replicon with stable cell lines generated by standard molecular biology techniques.

### Statistics

Target enrollment was approximately 285 patients. With this sample size, a response rate of 50 % in the control arm and 75 % in the vaniprevir arms would result in 95 % power to demonstrate that vaniprevir is superior to the control treatment at an alpha level of 0.05, as measured by the proportion of patients achieving SVR_24_.

The full analysis set population served as the primary population for the analysis of efficacy data and consisted of all randomized patients who received one or more doses of study treatment. Patients were included in the treatment group to which they were randomized for the analysis. For primary and secondary efficacy end points, differences between each vaniprevir arm and the control arm were assessed with use of 95 % confidence intervals (CIs) and associated *p* values calculated by the Miettinen and Nurminen method [18]. The all-patients-as-treated population was used for the analysis of safety data and consisted of all randomized patients who received one or more doses of study treatment and had at least one safety assessment; however, unlike the full analysis set population, patients were included in the treatment group that corresponded to the treatment they actually received for the analysis. AEs (specific terms as well as system organ class terms) were summarized by treatment arm. Safety end points prespecified as events of interest were subject to inferential testing for statistical significance with *p* values, and 95 % CIs provided between-group comparisons by the Miettinen and Nurminen method [18]. Summary statistics for baseline, after treatment, and change from baseline values were provided for laboratory parameters, 12-lead ECG, and vital signs.

## Results

**Fig. 1:**
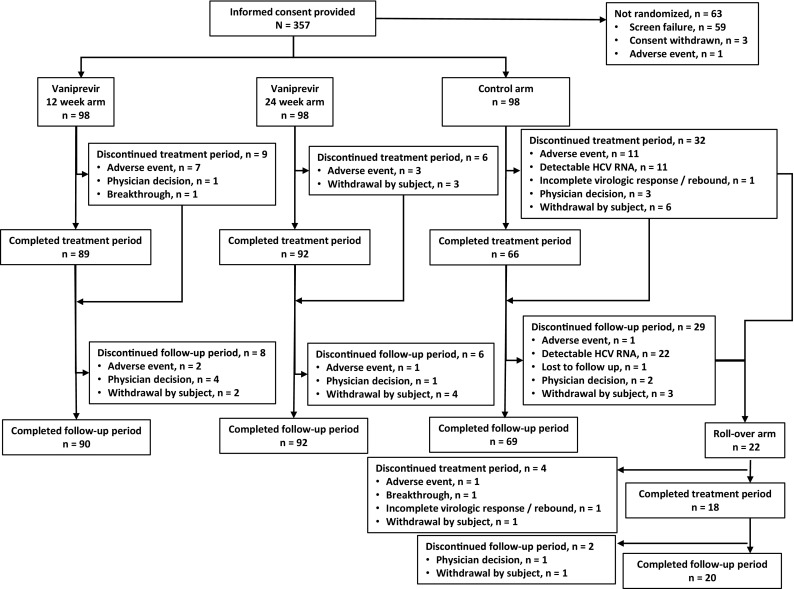
Study disposition. *HCV* hepatitis C virus

This study was performed at 55 study sites in Japan between July 2011 and March 2014. A total of 357 patients provided informed consent, and 294 were randomly assigned to treatment (98 patients in each arm). Fifteen patients randomly assigned to receive vaniprevir discontinued the treatment period (12-week arm, *n* = 9; 24-week arm, *n* = 6), and 14 patients discontinued the follow-up period (12-week arm, *n* = 8; 24-week arm, *n* = 6) (Fig. 1). In the control arm, 32 patients discontinued the treatment period, and 29 patients discontinued the follow-up period. Twenty-two patients with virologic failure in the control arm were enrolled in the rollover arm.

Patient demographics are shown in Table 1. No significant difference in demographic characteristics was observed between each vaniprevir arm and the control arm. The proportion of females was slightly higher in the vaniprevir 12-week arm than in the other arms, and the proportion of patients aged 65 years or older was similar across the three arms. Most patients had HCV genotype 1b infection and *IL28B* (rs12979860) CC genotype. All patients with *IL28B* (rs12979860) CC genotype had *IL28B* (rs8099917) TT genotype and five patients with *IL28B* (rs8099917) TT genotype had *IL28B* (rs12979860) CT genotype.

**Table 1:** Patient demographics

	Vaniprevir 12-week arm (*n* = 98)	Vaniprevir 24-week arm^a^ (*n* = 98)	Control arm (*n* = 98)	Total (*n* = 294)
Sex				
Male	42 (42.9 %)	49 (50.0 %)	46 (46.9 %)	137 (46.6 %)
Female	56 (57.1 %)	49 (50.0 %)	52 (53.1 %)	157 (53.4 %)
Age ≥65 years	15 (15.3 %)	17 (17.3 %)	16 (16.3 %)	48 (16.3 %)
HCV genotype				
1a	2 (2.0 %)	3 (3.1 %)	1 (1.0 %)	6 (2.0 %)
1b	96 (98.0 %)	95 (96.9 %)	97 (99.0 %)	288 (98.0 %)
Baseline HCV RNA (log_10_ IU/mL), mean ± SD	6.4 ± 0.6	6.5 ± 0.6	6.5 ± 0.7	6.5 ± 0.6
*IL28B* (rs12979860)				
CC	64 (65.3 %)	67 (68.4 %)	67 (68.4 %)	198 (67.3 %)
CT	32 (32.7 %)	30 (30.6 %)	30 (30.6 %)	92 (31.3 %)
TT	2 (2.0 %)	1 (1.0 %)	1 (1.0 %)	4 (1.4 %)
*IL28B* (rs8099917)				
TT	66 (67.3 %)	67 (68.4 %)	70 (71.4 %)	203 (69.0 %)
TG	31 (31.6 %)	30 (30.6 %)	27 (27.6 %)	88 (29.9 %)
GG	1 (1.0 %)	1 (1.0 %)	1 (1.0 %)	3 (1.0 %)
Neutrophils (10^2^/µL), mean ± SD	27.0 ± 8.3	29.4 ± 10.5	28.1 ± 9.5	28.2 ± 9.5
Hemoglobin (g/dL), mean ± SD	14.1 ± 1.3	14.4 ± 1.2	14.1 ± 1.2	14.2 ± 1.2
Platelets (10^4^/µL), mean ± SD	19.2 ± 5.1	18.0 ± 5.0	19.0 ± 5.1	18.8 ± 5.1
ALT (IU/L), mean ± SD	58.4 ± 48.3	57.9 ± 38.4	53.5 ± 40.9	56.6 ± 42.7
AST (IU/L), mean ± SD	48.1 ± 30.1	48.8 ± 25.9	45.8 ± 33.5	47.6 ± 30.0
Total bilirubin (mg/dL), mean ± SD	0.8 ± 0.3	0.8 ± 0.3	0.8 ± 0.3	0.8 ± 0.3

No significant difference in demographic characteristics was observed between each vaniprevir arm and the control arm

*ALT* alanine transaminase, *AST* aspartate transaminase, *HCV* hepatitis C virus, *SD* standard deviation

^a^One patient in the 24-week arm was excluded from the analysis for efficacy and safety as a result of receiving incorrect study medications; it was considered that appropriate evaluation for both efficacy and safety would not be possible

Four patients received incorrect study medications. Of these patients, one in the 24-week arm was excluded from the efficacy and safety analyses because it was considered that appropriate evaluation for both efficacy and safety was not possible. The other three patients were included in full analysis set and safety evaluation. These patients were included in their planned treatment groups for the efficacy analysis and in their actual treatment groups for safety and viral resistance analysis.

### Virologic response

The proportions of patients with SVR_24_ (the primary end point) were 83.7, 84.5, and 55.1 % in the vaniprevir 12-week, vaniprevir 24-week, and control arms, respectively. The difference in SVR_24_ rates between each vaniprevir arm and the control arm was statistically significant (*p* < 0.001 for both) (Table 2). The adjusted between-group differences (compared with the control arm) were 29.0 % (95 % CI 17.2–40.5) and 28.6 % (95 % CI 17.4–40.0) in the vaniprevir 12-week and vaniprevir 24-week arms, respectively. The rate of undetectable HCV RNA at treatment weeks 4 and 12 was significantly higher in each vaniprevir arm compared with the control arm (*p* < 0.001 for both). End-of-treatment responses were 95.9, 97.9, and 79.6 % in the vaniprevir 12-week, vaniprevir 24-week, and control arms, respectively (*p* < 0.001 for either vaniprevir treatment arm versus the control arm). Virologic failure was reported in 9.2 and 10.3 % of patients in the vaniprevir 12-week and vaniprevir 24-week arms, respectively. This was predominantly due to relapse, apart from one patient in the vaniprevir 12-week arm who had virologic breakthrough. In the control arm, virologic failure occurred in 38.8 % of patients. Relapse was commoner among patients in the control arm (29.5 %) than in those receiving vaniprevir (8.6 and 10.5 % for the 12- and 24-week arms, respectively).

**Table 2:** Virologic response rates

	Vaniprevir 12-week arm (*n* = 98)	Vaniprevir 24-week arm (*n* = 97)	Control arm (*n* = 98)
SVR_24_, all	82/98 (83.7 %)	82/97 (84.5 %)	54/98 (55.1 %)
SVR_24_ by subgroup			
Age (years)			
<65	71/83 (85.5 %)	68/80 (85.0 %)	47/82 (57.3 %)
≥65	11/15 (73.3 %)	14/17 (82.4 %)	7/16 (43.8 %)
*IL28B* (rs12979860)			
CC	59/64 (92.2 %)	63/66 (95.5 %)	46/67 (68.7 %)
CT/TT	23/34 (67.6 %)	19/31 (61.3 %)	8/31 (25.8 %)
Vaniprevir treatment adherence (% dosage received)			
<80 %	1/7 (14.3 %)	2/7 (28.6 %)	NA
≥80 %	81/91 (89.0 %)	80/90 (88.9 %)	NA
Peginterferon alfa-2b treatment adherence (% dosage received)			
<80 %	8/17 (47.1 %)	9/15(60.0 %)	6/37(16.2 %)
≥80 %	74/81 (91.4 %)	73/82 (89.0 %)	48/61 (78.7 %)
Ribavirin treatment adherence (% dosage received)			
<80 %	11/22 (50.0 %)	10/17(58.8 %)	15/45(33.3 %)
≥80 %	71/76 (93.4 %)	72/82 (90.0 %)	39/53 (73.6 %)
Virologic failure			
Breakthrough	1/98 (1.0 %)	0/97 (0 %)	0/98 (0 %)
Incomplete virologic response/rebound	0/98 (0 %)	0/97 (0 %)	5/98 (5.1 %)
Relapse	8/98 (8.2 %)	10/97 (10.3 %)	23/98 (23.5 %)
Detectable HCV RNA at TW36	NA	NA	10/98 (10.2 %)
Virologic response			
Undetectable HCV RNA at TW4	85/98 (86.7 %)	83/97 (85.6 %)	9/98 (9.2 %)
Undetectable HCV RNA at TW12	93/98 (94.9 %)	94/97 (96.9 %)	46/98 (46.9 %)
End-of-treatment response	94/98 (95.9 %)	95/97 (97.9 %)	78/98 (79.6 %)
SVR at follow-up week 12	82/98 (83.7 %)	82/97 (84.5 %)	53/98 (54.1 %)
Relapse after treatment completion			
Relapse rate	8/93 (8.6 %)	10/95 (10.5 %)	23/78 (29.5 %)

*HCV* hepatitis C virus, *NA* not applicable, *SVR* sustained virologic response, *SVR*_*24*_ sustained virologic response 24 weeks after completion of treatment, *TW* treatment week

### SVR_24_ subgroup analyses

In general, higher SVR_24_ rates were observed in the vaniprevir arms than in the control arm for patients younger than 65 years and for patients aged 65 years or older (Table 2). The adjusted between-group differences (compared with the control arm) were 28.2 % (95 % CI 14.8–40.99) and 27.7 % (95 % CI 14.0–40.56) in the vaniprevir 12-week and vaniprevir 24-week arms, respectively, for patients younger than 65 years and 29.6 % (95 % CI −5.7 to 58.27) and 38.6 % (95 % CI 5.4–64.62) in the vaniprevir 12-week and vaniprevir 24-week arms, respectively, for patients aged 65 years or older.

In all treatment arms, patients with the *IL28B* CT/TT allele tended to have lower SVR_24_ rates than patients with the *IL28B* CC allele (Table 2). Among patients with the *IL28B* CC allele, the differences in SVR_24_ rates between the vaniprevir and control arms were 23.5 and 26.8 % for the 12- and 24-week arms, respectively. Among patients with the *IL28B* CT/TT allele, the differences were 41.8 and 35.5 % in the 12- and 24-week arms, respectively. Of the six patients with HCV genotype 1a infection enrolled in the study, five patients were in the vaniprevir treatment groups (12-week arm, *n* = 2; 24-week arm, *n* = 3). Of these five patients, two achieved SVR_24_, two discontinued the study before follow-up week 24, and one relapsed.

Approximately 90 % of patients receiving vaniprevir (12-week arm and 24-week arm combined) were 80 % or more adherent to vaniprevir treatment and 78–85 % of patients were 80 % or more adherent to peginterferon alfa-2b or ribavirin treatment. Patients who were 80 % or more adherent to vaniprevir, peginterferon alfa-2b, or ribavirin treatment tended to have higher SVR_24_ rates than those who were less than 80 % adherent (Table 2).

### HCV RNA decline

The mean decline in HCV RNA levels was more rapid among patients in the vaniprevir arms than among those in the control arm (Fig. S1). Among patients in the vaniprevir arms, there was an approximate 5 log_10_ drop in mean HCV RNA levels during the first week of therapy. Overall, approximately 86 % of patients in the vaniprevir arms had undetectable HCV RNA at treatment week 4.

### Resistance-associated variants

#### Variants in the HCV NS3 gene

Baseline sequences were available for all patients who received vaniprevir; however, data from the one patient excluded from the efficacy and safety analyses were also excluded from the RAV analysis. In total, 127 of 195 patients treated with vaniprevir (65.1 %) had RAVs at the baseline, including 111 of 164 patients (67.7 %) who achieved SVR_24_ (Table 3). SVR_24_ was achieved by 111 of 127 patients (87.4 %) with RAVs at the baseline and 53 of 68 patients (77.9 %) without detectable RAVs at the baseline. Q80L (*n* = 21, including four patients with a Q/L mixed population), V170I (*n* = 83, including four patients with a V170I/M/V mixed population and three patients with a V170I/V mixed population), and Y56F (*n* = 66, including six patients with a Y56F/Y mixed population) were the commonest variants at the baseline, and no apparent difference in prevalence was observed between the SVR and non-SVR populations. In vitro, Q80L confers an eightfold loss of potency on vaniprevir, whereas Y56F and V170I confer potency losses of threefold or less (Table S1). Q80L was not associated with treatment failure, with 19 of 21 patients with baseline Q80L or Q80L/Q achieving SVR_24_ (Table 3). Greater in vitro potency losses due to vaniprevir result from mutations at R155, A156 (excluding A156S), or D168 (40-fold to several hundredfold; Table S1). Mutations at these residues were not detected at the baseline, with the exception of five patients with D168E or D168E/D mutations (40-fold potency shift in genotype 1b, Table S1), all of whom achieved SVR_24_ (Table 3).

**Table 3:** Distribution of baseline resistance-associated variants (RAVs) in the hepatitis C virus nonstructural protein 3 (NS3) region among patients receiving vaniprevir-based treatment

	Patients with SVR_24_		Patients with non-SVR_24_				Total	
			Meeting virologic failure criteria		Others			
	12-week arm (*n* = 82)	24-week arm (*n* = 82)	12-week arm (*n* = 9)	24-week arm (*n* = 10)	12-week arm (*n* = 7)	24-week arm (*n* = 5)	12-week arm (*n* = 98)	24-week arm (*n* = 97)
Patients with sample sequenced	82	82	9	10	7	5	98	97
Patients with any mutation	57/82 (69.5 %)	54/82 (65.9 %)	5/9 (55.6 %)	6/10 (60.0 %)	3/7 (42.9 %)	2/5 (40.0 %)	65/98 (66.3 %)	62/97 (63.9 %)
Patients with a specific mutation^a^								
V36L	1 (1.8 %)	0 (0 %)	0 (0 %)	0 (0 %)	0 (0 %)	0 (0 %)	1 (1.5 %)	0 (0 %)
Q41T	0 (0 %)	1 (1.9 %)	0 (0 %)	0 (0 %)	0 (0 %)	0 (0 %)	0 (0 %)	1 (1.6 %)
T54S	2 (3.5 %)	4 (7.4 %)	0 (0 %)	1 (16.7 %)	0 (0 %)	0 (0 %)	2 (3.1 %)	5 (8.1 %)
Y56F	27 (47.4 %)	25 (46.3 %)	3 (60.0 %)	0 (0 %)	3 (100 %)	2 (100 %)	33 (50.8 %)	27 (43.5 %)
Y56F/Y	3 (5.3 %)	3 (5.6 %)	0 (0 %)	0 (0 %)	0 (0 %)	0 (0 %)	3 (4.6 %)	3 (4.8 %)
Q80L	8 (14.0 %)	8 (14.8 %)	0 (0 %)	1 (16.7 %)	0 (0 %)	0 (0 %)	8 (12.3 %)	9 (14.5 %)
Q80L/Q	1 (1.8 %)	2 (3.7 %)	0 (0 %)	1 (16.7 %)	0 (0 %)	0 (0 %)	1 (1.5 %)	3 (4.8 %)
D168E	1 (1.8 %)	2 (3.7 %)	0 (0 %)	0 (0 %)	0 (0 %)	0 (0 %)	1 (1.5 %)	2 (3.2 %)
D168D/E	1 (1.8 %)	1 (1.9 %)	0 (0 %)	0 (0 %)	0 (0 %)	0 (0 %)	1 (1.5 %)	1 (1.6 %)
V170I	34 (59.6 %)	30 (55.6 %)	3 (60.0 %)	5 (83.3 %)	3 (100 %)	1 (50.0 %)	40 (61.5 %)	36 (58.1 %)
V170I/V	0 (0 %)	3 (5.6 %)	0 (0 %)	0 (0 %)	0 (0 %)	0 (0 %)	0 (0 %)	3 (4.8 %)
V170I/M/V	2 (3.5 %)	1 (1.9 %)	1 (20.0 %)	0 (0 %)	0 (0 %)	0 (0 %)	3 (4.6 %)	1 (1.6 %)
V170T	0 (0 %)	1 (1.9 %)	0 (0 %)	0 (0 %)	0 (0 %)	0 (0 %)	0 (0 %)	1 (1.6 %)

*SVR*_*24*_ sustained virologic response 24 weeks after completion of treatment

^a^Expressed as a percentage of the total number of patients with any baseline NS3 RAVs

Nineteen of 195 patients (9.7 %) enrolled in one of the two vaniprevir arms met the criteria for virologic failure (Tables 3, 4). Of these, 18 patients experienced viral relapse and one had viral breakthrough. Sixteen patients had RAVs at failure (D168V, *n* = 9; D168D/V mixed population, *n* = 1; D168H, *n* = 1; D168T, *n* = 1; R155 K, *n* = 2; T54S, *n* = 1; Y56F, *n* = 2; Q80L, *n* = 3; V170I, *n* = 8). Two patients did not have any known RAVs at failure, and a sequence was unavailable for one patient.

**Table 4:** Patients in the vaniprevir arms who met the virologic failure criteria and resistance-associated variants (RAVs) in the hepatitis C virus (HCV) nonstructural protein 3 (NS3) region detected at the baseline, at failure, and during the follow-up period

Patient	Treatment group (week)	Treatment failure category	Genotype	*IL28B* (rs12979860)	Age (years)	Sex	Treatment failure confirmed (study day)^a^	RAVs in NS3 region at the baseline	Sample collection day for RAVs in NS3 region at failure^b^	RAVs in NS3 region					
										~FU4		FU12	FU20		FU24
1	12	Relapse^c^	1b	TT	59	F	199	Y56F, V170I	213 (FU4)	Y56F, D168V, V170I		Y56F, D168V, V170I	Y56F, D168D/V, V170I		Y56F, D168D/V, V170I
2	12	Relapse^c^	1a	CT	54	F	197	None	210 (FU4)	R155K		R155K	R155K		R155K
3	12	Relapse^c^	1b	CT	69	M	197	None	205 (FU4)	D168V		None	None		None
4	12	Virologic breakthrough^d^	1b	CT	55	M	140	Y56F	161 (on treatment)	Y56F, R155K		NC	NC		NC
5	12	Relapse^c^	1b	CC	64	F	57	Y56F, V170I/M/V	NC	NC		NC	NC		NC
6	12	Relapse^c^	1b	CT	61	F	252	V170I	259 (FU12)	NA		D168H, V170I	D168D/H, V170I		V170I
7	12	Relapse^c^	1b	CT	65	F	223	V170I	234 (FU4)	D168V, V170I		D168D/V, V170I	D168D/V, V170I		D168D/V, V170I
8	12	Relapse^c^	1b	CT	25	F	86	None	96 (FU4)	D168V		None	None		None
9	12	Relapse^c^	1b	CT	22	M	253	None	270 (FU12)	NA		None	None		None
10	24	Relapse^c^	1b	CT	63	F	203	None	259 (FU12)	NA		Q80L	NC		Q80L
11	24	Relapse^c^	1b	CT	62	F	205	None	211 (FU4)	Q80L, D168V		D168V	D168V		D168V
12	24	Relapse^c^	1b	CT	60	F	204	None	260 (FU12)	NA		D168T	D168T		D168A/D/N/T
13	24	Relapse^c^	1b	CT	59	M	196	V170I	203 (FU4)	D168V, V170I		D168D/V, V170I	V170I		V170I
14	24	Relapse^c^	1b	CT	56	F	253	Q80L/Q	316 (FU20)	NA		NA	None		None
15	24	Relapse^c^	1b	CT	60	F	253	V170I	260 (FU12)	NA		D168V, V170I	V170I		V170I
16	24	Relapse^c^	1b	CT	43	F	205	V170I	213 (FU4)	D168V, V170I		D168V, V170I	D168D/V, V170I		V170I
17	24	Relapse^c^	1b	CT	66	M	197	None	225 (FU4)	D168V		D168V	NC		NC
18	24	Relapse^c^	1b	CT	67	F	246	T54S, Q80L, V170I	373^e^	NC		NC	NC		T54S, Q80L, V170I^e^
19	24	Relapse^c^	1b	CT	55	F	197	V170I	209 (FU4)	D168D/V, V170I		V170I		V170I	V170I

These variants encompass amino acid residues 36, 41, 43, 54, 55, 56, 80, 155, 156, 168, and 170 in the NS3 region

*F* female, *FU* follow-up week, *M* male, *NC* not collected, *NA* not applicable

^a^The study day is the time of the first visit for two consecutive visits

^b^At failure includes the testing result obtained with the samples collected at failure, or if they are unavailable the next available sample

^c^Any patient who has two consecutive visits with detectable HCV RNA following the end of all study treatment, after HCV RNA became undetectable while the patient was receiving treatment. The second visit would be an unscheduled visit within 2 weeks of the first visit

^d^Any patient who had undetectable HCV RNA and subsequently had an HCV RNA level greater than 1000 IU/mL while receiving therapy

^e^Samples at failure (FU4), FU12, and FU20 were not collected, and testing results at FU24 are only available for the patient after the baseline

Virologic failure was principally associated with the emergence of mutations at D168 or R155, with 14 patients (73.7 %) having a mutation emerge at failure at one of these two loci. In all cases these mutations were not present at the baseline but emerged during treatment. The D168 mutations diminished rapidly following completion or discontinuation of vaniprevir treatment as evidenced by the reappearance of wild-type virus at follow-up visits. The T54S, Y56F, and V170I variants observed at failure were also observed at the baseline, and did not emerge during therapy.

Of the two patients with the R155K variant at failure, one had HCV genotype 1a infection (relapse) and one had HCV genotype 1b infection (breakthrough). The R155K variant is uncommon in patients with HCV genotype 1b infection because a two-nucleotide change is necessary to generate the mutation. Sequencing of a baseline sample from this patient revealed a common HCV genotype 1b codon for R155, indicating that the R155K variant emerged from a rare two-nucleotide mutation. The patient with HCV genotype 1b infection and the R155K variant at failure discontinued participating in the study, and there were no additional results following the time of failure. For the patient with HCV genotype 1a infection and the R155K variant at failure, R155K was continuously detected through to the patient’s final visit, which was conducted 24 weeks after completion of treatment.

Three patients had a Q80L mutation at failure. For one of these patients, D168V was also detected at failure; neither mutation was detected at the baseline, and the emerging D168V mutation (which confers a significant potency loss on vaniprevir) is the likely cause of virologic failure. Another patient had a combination of T54S, Q80L, and V170I mutations at both the baseline and failure. Subsequent phenotyping in the replicon assay demonstrated that the triple combination of T54S:Q80L:V170I conferred a negligible change in vaniprevir potency (Table S1), either when engineered into a genotype 1b reference strain or as a chimeric replicon matching this patient’s NS3 protease sequence. The third patient had only a Q80L mutation at failure, but this was not observed at the baseline. As discussed earlier, Q80L was commonly observed at the baseline among patients who achieved SVR_24_ and does not appear to be linked to virologic failure of vaniprevir treatment. In addition to these three patients, there was one additional patient who had no known RAVs at failure despite the presence of the Q80L variant at the baseline. This patient had a Q80L/Q mixed population at the baseline, but was homogeneous for Q80 at failure.

#### Variants in the HCV NS5A gene

A total of 169 samples collected at the baseline from patients who consented to optional specimen collection for future biomedical research were sequenced through the HCV NS5A gene and were available for analysis. Table S2 shows the distribution of the polymorphisms within the NS5A gene at the baseline that were detected at positions L31 and Y93, positions strongly linked to NS5A inhibitor resistance, and those which were detected at other positions in 10 % or more of patients in one or more treatment groups. The commonest of these latter variants were F37L (85/147, 57.8 %) and Q54H (64/147, 43.5 %). Generally, no apparent difference in prevalence of these NS5A variants was observed between SVR and non-SVR populations. Mutations at L31 were detected only in patients who achieved SVR_24_. Of the mutations detected at L31, the prevalence of baseline L31M was 2.4 % (four of 169 patients). The prevalence of Y93H mutations (including Y93Y/H and Y93Y/C/H) was 16.0 % (27 of 169 patients). Y93Y/F was detected in one patient who achieved SVR_24_. The SVR_24_ rate in patients with these baseline Y93 mutations was 75.0 % (six of eight patients) and 84.6 % (11 of 13 patients) in the vaniprevir 12-week and vaniprevir 24-week arms, respectively, and was considered comparable with the overall SVR_24_ rate for each vaniprevir arm (83.7 and 84.5 % in the 12- and 24-week arms, respectively). There were no treatment-emerging mutations in NS5A in patients who met the criteria for virologic failure and for whom the results of NS5A sequencing were available (Table S3), and the outcome from vaniprevir-based treatment was not influenced by the baseline presence of major NS5A mutations, as expected from the mode of action of vaniprevir, which targets the NS3 protease.

### Safety

The AE profile was largely similar across all treatment arms. Administration of vaniprevir did not increase the incidence of SAEs or discontinuations due to AEs relative to treatment with PR alone (Table 5). No deaths were reported. Commonly reported AEs were generally similar in the vaniprevir arms and the control arm, with the exception of an increase in the incidence of gastrointestinal AEs such as vomiting, nausea, and diarrhea in the vaniprevir arms compared with the control arm. Gastrointestinal AEs (vomiting, nausea, and diarrhea) occurred more frequently in the vaniprevir 12-week arm than in the control arm (62.2 % vs 46.9 %, *p* = 0.032); however, no significant difference in frequency was observed between the vaniprevir 24-week arm and the control arm (52.6 % vs 46.9 %, *p* = 0.432). These gastrointestinal AEs (vomiting, nausea, and diarrhea) tended to occur early during the course of therapy (approximately within 2 weeks after the start of treatment). One patient receiving vaniprevir for 12 weeks had a gastrointestinal SAE of moderate vomiting, which resolved on treatment after a ribavirin dose reduction, and two patients discontinued use of the study medications because of gastrointestinal AEs (moderate vomiting in one patient in the 12-week arm, and severe vomiting and diarrhea in one patient in the 24-week arm). One additional patient in the 12-week arm reported severe nausea. All other gastrointestinal AEs were mild to moderate in severity. Thus, gastrointestinal AEs were deemed manageable.

**Table 5:** Adverse events (AEs)

	Vaniprevir 12-week arm (*n* = 98)	Vaniprevir 24-week arm (*n* = 97)	Control arm (*n* = 98)
Any AE	98 (100.0 %)	97 (100.0 %)	98 (100.0 %)
Serious AEs,	5 (5.1 %)	6 (6.2 %)	9 (9.2 %)
Serious drug-related AEs	4^a^ (4.1 %)	4^b^ (4.1 %)	4^c^ (4.1 %)
Deaths	0 (0.0 %)	0 (0.0 %)	0 (0.0 %)
Discontinuation due to an AE	7 (7.1 %)	3 (3.1 %)	11 (11.2 %)
Discontinuation due to a drug-related AE	7^d^ (7.1 %)	3^e^ (3.1 %)	10^f^ (10.2 %)
Common AEs^g^			
Pyrexia	79 (80.6 %)	69 (71.1 %)	80 (81.6 %)
Neutrophil count decreased	50 (51.0 %)	46 (47.4 %)	43 (43.9 %)
Headache	49 (50.0 %)	47 (48.5 %)	46 (46.9 %)
White blood cell decreased	45 (45.9 %)	44 (45.4 %)	45 (45.9 %)
Rash	42 (42.9 %)	33 (34.0 %)	45 (45.9 %)
Nausea	36 (36.7 %)	32 (33.0 %)	27 (27.6 %)
Hemoglobin decreased	35 (35.7 %)	31 (32.0 %)	42 (42.9 %)
Decreased appetite	32 (32.7 %)	32 (33.0 %)	35 (35.7 %)
Malaise	31 (31.6 %)	32 (33.0 %)	37 (37.8 %)
Alopecia	31 (31.6 %)	30 (30.9 %)	33 (33.7 %)
Arthralgia	30 (30.6 %)	34 (35.1 %)	29 (29.6 %)
Diarrhea	30 (30.6 %)	21 (21.6 %)	22 (22.4 %)
Pruritus	29 (29.6 %)	34 (35.1 %)	35 (35.7 %)
Platelet count decreased	28 (28.6 %)	36 (37.1 %)	36 (36.7 %)
Vomiting	25 (25.5 %)	30 (30.9 %)	9 (9.2 %)
Nasopharyngitis	21 (21.4 %)	29 (29.9 %)	31 (31.6 %)
AEs of interest^h^			
Any event	88 (89.8 %)	81 (83.5 %)	84 (85.7 %)
Anemia/hemoglobin decreased	59 (60.2 %)	50 (51.5 %)	63 (64.3 %)
Bilirubin increased	7 (7.1 %)	12 (12.4 %)	7 (7.1 %)
Gastrointestinal AEs (vomiting, nausea, diarrhea)	61 (62.2 %)^i^	51 (52.6 %)	46 (46.9 %)
Neutropenia/neutrophil decreased	58 (59.2 %)	50 (51.5 %)	50 (51.0 %)

*AE* adverse event

^a^Inappropriate antidiuretic hormone secretion, vomiting, decreased appetite, diabetes mellitus

^b^Chondrocalcinosis pyrophosphate, hepatic function abnormal, atrial fibrillation and dehydration, hepatocellular carcinoma

^c^Gastric cancer, sudden hearing loss, endolymphatic hydrops, fatigue

^d^Inappropriate antidiuretic hormone secretion, fatigue and decreased appetite, fatigue and dizziness, hemoglobin decreased, vomiting, decreased appetite, diabetes mellitus

^e^Hepatic function abnormal, diarrhea and vomiting, peripheral neuropathy

^f^Blood alkaline phosphatase increased and gammaglutamyltransferase increased, depressed mood, gastric cancer, sudden hearing loss, retinopathy, anemia, interstitial lung disease, anxiety, anemia, gingival swelling, nausea

^g^Incidence greater than 30 % in any treatment arm

^h^No patients had serious rash

^i^ *p* = 0.032 versus the control

The incidence of anemia, blood bilirubin increased level, and neutropenia was similar across treatment arms, and serious rash was not reported in any patient (Table 5). In addition, mean changes from the baseline in the laboratory tests for hemoglobin, bilirubin, and neutrophils were not different between the vaniprevir arms and the control arm. A similar trend was observed in alanine transaminase level, aspartate aminotransferase level, and platelet count (Fig. S2). There were no clinically meaningful differences in vital signs or in ECG parameters between the vaniprevir arms and the control arm. Overall, the safety profiles were comparable between the 12- and 24-week arms.

### Rollover arm

Of the 22 patients with virologic failure who were enrolled in the rollover arm, four patients discontinued use of the study medications (Fig. 1) (one each because of an AE of decreased appetite, virologic breakthrough, incomplete virologic response/rebound, and withdrawal by the patient). The efficacy, viral resistance, and safety outcomes in the rollover arm were consistent with the results from other vaniprevir studies in treatment-experienced patients.

## Discussion

In the present study, the addition of vaniprevir treatment to PR treatment was associated with a significant increase in SVR_24_ rates compared with PR treatment alone in treatment-naive, noncirrhotic Japanese patients with HCV genotype 1 infection. Both vaniprevir regimens evaluated in this study had a 24-week duration, in contrast to the standard 48-week duration for PR treatment alone. Overall, approximately 86 % of patients in the vaniprevir arms had undetectable HCV RNA at treatment week 4, and approximately 84 % achieved SVR_24_ at 24 weeks after completion of therapy. On-treatment virologic failure was uncommon, with only one breakthrough reported in the vaniprevir 12-week arm. Nearly all patients treated with vaniprevir (97 %) had an end-of-treatment response. Most of the vaniprevir recipients in whom treatment failed relapsed after completion of therapy. The increased response rates for vaniprevir-based therapy relative to PR therapy alone remained consistent across major patient subgroups, including those with *IL28B* CC and CT/TT genotypes and those younger than 65 years and those aged 65 years or older. Interpretation of efficacy differences according to HCV genotype is difficult because few Japanese patients with genotype 1a infection were enrolled in this study. In total, 98 % of patients in the present study had HCV genotype 1b infection. However, a previous phase II study of non-Japanese patients reported significantly higher SVR_24_ rates in patients with HCV genotype 1a infection (41.7 % of the study population) who received vaniprevir-based therapy compared with those receiving PR therapy alone (56.3–83.3 vs 20 %) [15]. Therefore, the efficacy of vaniprevir-based triple therapy in patients with HCV genotype 1a infection is expected. In this study, a total of five patients with *IL28B* (rs8099917) TT genotype had *IL28B* (rs12979860) CT genotype. Therefore, five patients (1.7 %, five of 294 patients) had SNPs (rs12979860 and rs8099917) not in linkage disequilibrium. This finding is similar to that reported by Ito et al. [19], in which 98.6 % of cases were in linkage disequilibrium for the four different SNPs analyzed (rs11881222, rs8103142, rs12979860, and rs8099917).

In this study, virologic failure was principally associated with the emergence of mutations at D168, specifically D168H, D168T, or D168V, and to a lesser extent at R155. These mutations were not detected at the baseline but rather emerged during vaniprevir treatment. D168 mutants rapidly disappeared during the follow-up period, with nine of 12 patients with a D168 mutation in whom treatment failed showing diminished levels of mutant virus concomitant with increased levels of wild-type virus during the follow-up period; the D168 mutation became undetectable in seven of these patients. R155K persistence could not be adequately addressed in this study as there were only two patients with this mutation in whom treatment failed: one patient with HCV genotype 1a infection with continuous detection through to the patient’s final visit 24 weeks after treatment completion and a patient with HCV genotype 1b infection who discontinued participating in the study and for whom there were no additional results following the time of failure. The presence of other variants at the baseline that are associated with failure of some DAA regimens did not appear to affect the outcome of vaniprevir-based treatment. This is evidenced by the similar SVR rates in patients with these variants at the baseline compared with patients with wild-type virus at the baseline, and is also supported by in vitro replicon data confirming that mutations at residues other than R155, A156, or D168 have at most a modest impact on vaniprevir potency. It is also noted that five patients were found to have D168E (including D168D/E mixtures) at the baseline, all of whom achieved SVR_24_. D168E (40- to 58-fold potency shift, Table S1) was not noted among any of the patients in whom vaniprevir-based treatment failed (Table S3), and the 40-fold loss associated with D168E may suggest a threshold measurement of the potency loss necessary before failure is a concern.

Variants in the HCV NS5A region are not anticipated to affect the efficacy of vaniprevir because the NS3 protease, not the NS5A gene product, is the drug target. Consistent with this, mutations in NS5A at loci linked to drug resistance do not impact vaniprevir potency in vitro (Table S1). However, in consideration of the Japan Society of Hepatology “Guidelines for the management of hepatitis C virus infection,” the presence of NS5A variants at the baseline is increasingly becoming of interest within interferon-free, all-DAA regimens. Consistent with this approach, it has previously been reported that L31M and Y93H mutations were detected at the baseline (by means of direct sequencing methods) in approximately 3.3 % of Japanese patients (seven of 214 patients) and 14.0 % of Japanese patients (30 of 214 patients) enrolled in the daclatasvir and asunaprevir Japanese registration study [10]. In the present study, the prevalences of L31M and Y93H were similar to those in previously reported studies. The distribution of mutations commonly detected at the baseline was not notably different between SVR and non-SVR populations. Furthermore, there were no treatment-emerging mutations in NS5A among patients in whom treatment failed (Table S3). In the small number of patients with virologic failure in the present study, the virologic cause of failure is solely attributable to mutations within the NS3/4A region, resulting in decreased vaniprevir potency.

The incidence of AEs was similar in patients in the vaniprevir arms and in patients in the control arm. There was no serious rash among patients receiving vaniprevir, the frequency of SAEs and discontinuations due to AEs were similar across all treatment arms, and there were no specific trends in SAEs or discontinuations due to AEs. Among the commonest AEs (incidence of 30 % or more in one or more arms), only vomiting was reported at an incidence of 10 % or higher in the vaniprevir arms compared with the control arm. In the analysis of prespecified events of interest, the incidence of the grouped gastrointestinal AEs, vomiting, nausea, and diarrhea, was significantly higher in the vaniprevir 12-week arm than in the control arm (62.2 % vs 46.9 %, *p* = 0.032), primarily driven by the difference in the rates of vomiting between these treatment arms. Overall, these observations are consistent with data from previous studies of vaniprevir [14–17]. These gastrointestinal AEs are considered manageable given that they tended to develop early after the start of treatment and in almost all instances recovered without the need for treatment discontinuation. The efficacy/safety profile of vaniprevir plus PR in Japanese patients is therefore consistent with previous reports in non-Japanese patients [14–16].

In conclusion, the results of this phase III study demonstrate that the addition of vaniprevir treatment to PR treatment results in a significant increase in SVR_24_ rates compared with PR treatment alone. On the basis of results of this study and parallel studies in patients with previous treatment failure (Kumada et al., manuscript in preparation), vaniprevir plus PR has recently received marketing approval for the treatment of Japanese treatment-naive and treatment-experienced patients with HCV genotype 1 infection. The approved regimen for treatment-naive patients and patients who relapsed following prior interferon-based treatment is vaniprevir plus PR for 12 weeks followed by PR for an additional 12 weeks (total treatment duration 24 weeks). The approved regimen for patients who were nonresponders to prior interferon-based treatment is vaniprevir plus PR for 24 weeks. Vaniprevir therefore provides a valuable addition to the therapeutic options for Japanese patients with HCV genotype 1 infection who are eligible for interferon-based treatment according to the Japan Society of Hepatology “Guidelines for the management of hepatitis C virus infection.”

## Electronic supplementary material

Supplementary Tables (DOCX 59.7 kb)

Fig. S1 Mean hepatitis C virus (*HCV*) RNA level decrease from the baseline (full analysis set) (EPS 1538 kb)

Fig. S2a Change from baseline laboratory observations during the treatment period and the follow-up period: alanine aminotransferase (*ALT*). *FU* follow-up week (EPS 1647 kb)

Fig. S2b Change from baseline laboratory observations during the treatment period and the follow-up period: aspartate aminotransferase *(AST*). *FU* follow-up week (EPS 1658 kb)

Fig. S2c Change from baseline laboratory observations during the treatment period and the follow-up period: total bilirubin. *FU* follow-up week (EPS 1661 kb)

Fig. S2d Change from baseline laboratory observations during the treatment period and the follow-up period: hemoglobin. *FU* follow-up week (EPS 1654 kb)

Fig. S2e Change from baseline laboratory observations during the treatment period and the follow-up period: platelets. *FU* follow-up week (EPS 1657 kb)

Fig. S2f Change from baseline laboratory observations during the treatment period and the follow-up period: neutrophils. *FU* follow-up week (EPS 1687 0 kb)
