# Supplementary material for: Vaniprevir plus peginterferon alfa-2b and ribavirin in treatment-naive Japanese patients with hepatitis C virus genotype 1 infection: a randomized phase III study
Source: J Gastroenterol. 2015 Sep 25;51:390–403. doi: 10.1007/s00535-015-1120-x (PMC4805724; doi:10.1007/s00535-015-1120-x)
Supplement: Supplementary file 1 — Supplementary Tables (DOCX 59.7 kb) [file 535_2015_1120_MOESM1_ESM.docx]

**Supplementary Table 1** Inhibitory potency of vaniprevir against panels of replicon mutants in the GT1a or GT1b backbones

Please include full description on incomplete virologic response in the box or explanation on abbreviation in the footnote.

| Genotype | EC_90_, nM | Fold-shift to wild type |
| --- | --- | --- |
| GT1a wild type (H77) | 3.4 ± 2.3 | 1 |
| NS3 |  |  |
| GT1a V36L | 4.7 ± 1.8 | 1.4 |
| GT1a Q41R | 4.7 ± 1.9 | 1.4 |
| GT1a F43L | 27.0 ± 11.0 | 7.9 |
| GT1a T54S | 4.7 ± 3.6 | 1.4 |
| GT1a Q80K | 4.5 ± 2.2 | 1.3 |
| GT1a R155K | >1000 | >294 |
| GT1a A156S | 17.0 ± 9.1 | 5.0 |
| GT1a D168E | 193 ± 213 | 57 |
| GT1a D168T | >2,000 | >588 |
| GT1a D168V | >2,000 | >588 |
| NS5A |  |  |
| GT1a Q30E | 4.9 ± 1.9 | 1.4 |
| GT1a Q30R | 7.3 ± 3.5 | 2.1 |
| GT1a L31V | 9.2 ± 3.5 | 2.7 |
| GT1a Y93H | 12.0 ± 4.6 | 3.5 |
| GT1a Y93N | 6.4 ± 4.4 | 1.9 |
| GT1a L31M:Y93H | 4.9 ± 2.3 | 1.4 |
| GT1b wild type (Con 1) | 4.9 ± 3.9 | 1 |
| NS3 |  |  |
| GT1b V36L | 2.8 ± 1.0 | 0.6 |
| GT1b Q41L | 2.2 ± 1.4 | 0.6 |
| GT1b Q41R | 5.1 ± 2.0 | 1.3 |
| GT1b T54S | 6.8 ± 2.0 | 1.4 |
| GT1b Y56F | 5.0 ± 1.5 | 1.0 |
| GT1b Q80L | 40.0 ± 21.1 | 8.2 |
| GT1b S122G | 3.4 ± 0.7 | 0.7 |
| GT1b S122R | 7.1 ± 0.9 | 1.4 |
| GT1b R155K | 945 ± 228 | 193 |
| GT1b R155Q | 649 ± 214 | 132 |
| GT1b A156S | 50.0 ± 20.9 | 10 |
| GT1b A156T | 400 ± 164 | 82 |
| GT1b D168E | 193 ± 82 | 40 |
| GT1b D168H | >2,000 | >408 |
| GT1b D168T | >2,000 | >408 |
| GT1b D168V | >2,000 | >408 |
| GT1b V170I | 14.0 ± 5.3 | 2.9 |
| GT1b V170T | 14.1 ± 7.8 | 2.9 |
| GT1b T54S:Q80L:V170I | 3.4 ± 0.8 | 0.7 |
| NS5A |  |  |
| GT1b L28M | 4.6 ± 1.4 | 0.9 |
| GT1b L28V | 2.4 ± 1.3 | 0.5 |
| GT1b R30Q | 4.0 ± 2.3 | 0.8 |
| GT1b L31M | 2.2 ± 1.3 | 0.4 |
| GT1b L31V | 4.2 ± 2.1 | 0.9 |
| GT1b Y93H | 4.1 ± 1.5 | 0.8 |
| GT1b L31M:Y93H | 2.0 ± 0.6 | 0.4 |

*EC_90_* 90% effective concentration, *GT* genotype, *NS* non-structural

**Supplementary Table 2** Distribution of baseline variants in HCV NS5A region among patients receiving vaniprevir-based treatment ^a^

|  | Patients with SVR_24_ | | | Patients with non-SVR_24_ | | | Total |
| --- | --- | --- | --- | --- | --- | --- | --- |
|  | 12-wk arm  (*n* = 82) | 24-wk arm  (*n* = 82) | Control arm  (*n* = 54) | 12-wk arm  (*n* = 16) | 24-wk arm  (*n* = 15) | Control arm  (n =44) |  |
| Patients with sample sequenced for NS5A region | 45 | 50 | 31 | 7 | 9 | 27 | 169 |
| Patients with any mutation | 39/45 (86.7) | 45/50 (90.0) | 24/31 (77.4) | 5/7 (71.4) | 9/9 (100) | 25/27 (92.6) | 147/169 (87.0) |
| Patients with specific mutation, *n* (%)^b^ | | | | | | | |
| L31I, L31L/I | 2/39 (5.1) | 0/45 (0) | 0/24 (0) | 0/5 (0) | 0/9 (0) | 0/25 (0) | 2/147 (1.4) |
| L31M | 2/39 (5.1) | 0/45 (0) | 2/24 (8.3) | 0/5 (0) | 0/9 (0) | 0/25 (0) | 4/147(2.7) |
| L31F | 0/39 (0) | 0/45 (0) | 1/24 (4.2) | 0/5 (0) | 0/9 (0) | 0/25 (0) | 1/147 (0.7) |
| Y93H, Y93Y/H, Y93Y/C/H | 5/39 (12.8) | 11/45 (24.4) | 3/24 (12.5) | 2/5 (40.0) | 2/9 (22.2) | 4/25 (16.0) | 27/147 (18.4) |
| Y93Y/F | 1/39 (2.6) | 0/45 (0) | 0/24 (0) | 0/5 (0) | 0/9 (0) | 0/25 (0) | 1/147 (0.7) |
|  |  |  |  |  |  |  |  |
| L28M, L28L/M | 3/39 (7.7) | 5/45 (11.1) | 1/24 (4.2) | 0/5 (0) | 1/9 (11.1) | 1/25 (4.0) | 11/147 (7.5) |
| R30Q, R30R/Q, R30L/Q | 6/39 (15.4) | 7/45 (15.6) | 3/24 (12.5) | 2/5 (40.0) | 1/9 (11.1) | 3/25 (12.0) | 22/147 (15.0) |
| F37L, F37F/L, F37I/L | 27/39 (69.2) | 20/45 (44.4) | 13/24 (54.2) | 3/5 (60.0) | 5/9 (55.6) | 17/25 (68.0) | 85/147 (57.8) |
| Q54H, Q54Q/H, Q54H/Y, Q54F/H/L, Q54Q/H/L | 14/39 (35.9) | 18/45 (40.0) | 9/24 (37.5) | 1/5 (20.0) | 5/9 (55.6) | 17/25 (68.0) | 64/147 (43.5) |
| A92T, A92A/T | 6/39 (15.4) | 1/45 (2.2) | 2/24 (8.3) | 0/5 (0) | 0/9 (0) | 1/25 (4.0) | 11/44 (7.5) |

*GT* genotype, *HCV* hepatitis C virus, *NS* non-structural, *RAV* resistance-associated variant, *SVR_24_* sustained virologic response 24 weeks after completing treatment

^a^Testing for variants in NS5A region was conducted using archived plasma samples collected from patients with GT 1b infection and who consented to optional specimen collection for future biomedical research. Variants at amino acid residues L23, Q24, L28, R30, L31, P32, F37, Q54, P58, Q62, A92, and Y93 were reported if detected. This table includes all mutations at L31 and Y93 and other mutations which were detected in >10% of patients in one or more treatment groups

^b^Expressed as a % of the total number of patients with any baseline NS5A RAV

**Supplementary Table 3** Listing of patients who met virologic failure criteria and had results of NS5A sequencing

| **Patients** | **Treatment** | **Treatment** | **GT** | **Age** | **Gender** | **Log_10_ of** | **MK-7009** | **NS5A** | **Treatment** | **Sample** | **MK-7009** | **Sample** | **NS5A** |
| --- | --- | --- | --- | --- | --- | --- | --- | --- | --- | --- | --- | --- | --- |
|  | **group** | **failure category** |  | **(years)** |  | **baseline HCV RNA** | **resistant NS3 mutations^a^ at baseline** | **mutations^b^ at baseline** | **failure confirmed (study day)^c^** | **collection date for NS3 mutations^a^ at failure^d^** | **resistant NS3 mutations^a^ at failure^d^** | **collection date for NS5A mutations^b^ at failure^d^** | **mutations^b^ at failure^d^** |
| 1 | 12 wk | RELAPSE^e^ | 1b | 59 | F | 5.7 | Y56F, V170I | None | 199 | 213 | Y56F, D168V,V170I, | 213 | None |
| 5 | 12 wk | RELAPSE^e^ | 1b | 64 | F | 7.1 | Y56F, V170I/M/V | F37L, Q54H/Y, Y93Y/H | 57 | Not collected | | | |
| 7 | 12 wk | RELAPSE^e^ | 1b | 65 | F | 5.8 | V170I | Q24K, L28V, R30Q | 223 | 234 | D168V,V170I | 234 | Q24K, L28V, R30Q |
| 9 | 12 wk | RELAPSE^e^ | 1b | 22 | M | 5.2 | None | None | 253 | 270 | None | NA | NA |
| 10 | 24 wk | RELAPSE^e^ | 1b | 63 | F | 6.5 | None | Q24K, L28M, R30Q | 203 | 259 | Q80L | 259 | Q24K, L28M, R30Q |
| 11 | 24 wk | RELAPSE^e^ | 1b | 62 | F | 6.5 | None | F37F/L, Q54H/Y | 205 | 211 | Q80L, D168V | 211 | Q54Y |
| 13 | 24 wk | RELAPSE^e^ | 1b | 59 | M | 6.4 | V170I | F37L | 196 | 203 | D168V,V170I | 203 | F37L |
| 15 | 24 wk | RELAPSE^e^ | 1b | 60 | F | 6.4 | V170I | F37L, Q54H | 253 | 260 | D168V,V170I | 260 | F37L, Q54H |
| 17 | 24 wk | RELAPSE^e^ | 1b | 66 | M | 6.4 | None | F37L | 197 | 225 | D168V | 225 | F37L |
| 19 | 24 wk | RELAPSE^e^ | 1b | 55 | F | 5.6 | V170I | Q54H, P58T | 197 | 209 | D168D/V,  V170I | 209 | Q54H, P58T |
| NA: Testing was not performed because of the lack of volume of archived plasma samples  ^a^These variants encompass amino acid residues 36, 41, 43, 54, 55, 56, 80, 155, 156, 168 and 170 in NS3 region  ^b^Testing for variants in NS5A region was conducted using archived plasma samples collected from patients with GT 1b infection and consent to optional specimen collection for future biomedical research. Variants in amino acid residues L23, Q24, L28, R30, L31, P32, F37, Q54, P58, Q62, A92 and Y93 were reported if detected  ^c^Confirmed study day was the time of the first visit in two consecutive visits  ^d^At failure also included the testing result using the available sample collected closest to the time of failure in addition to at failure  ^e^Any patient who had two consecutive visits with detectable HCV RNA following end of all study treatment, after becoming undetectable on treatment. The second visit would be an unscheduled visit within 2 weeks of the first visit | | | | | | | | | | | | | |

**Figure Legends**

**Supplementary Fig. 1** Mean HCV RNA decrease from baseline (FAS) *FAS* full analysis set, *HCV* hepatitis C virus

**Supplementary Fig. 2** Change from baseline laboratory observations during treatment period and follow-up period: **A** alanine aminotransferase, **B** aspartate aminotransferase, **C** total bilirubin, **D** hemoglobin, **E** platelets, **F** neutrophils

*BID* twice daily, *FU* follow-up week, *PR* peginterferon alfa-2b and ribavirin
